# A new species of *Austrocodrus* Ogloblin (Hymenoptera, Proctotrupidae, Austroserphinae), a Gondwanic relict from southernmost South America

**DOI:** 10.3897/zookeys.803.28360

**Published:** 2018-11-06

**Authors:** Enrique Rodríguez-Serrano, Alvaro Zúñiga-Reinoso

**Affiliations:** 1 Departamento de Zoología, Facultad de Ciencias Naturales y Oceanográficas, University of Concepción, Concepción, Casilla 160-C, Chile University of Concepción Concepción Chile; 2 Institut für Zoologie, University of Cologne, Zülpicher Str. 47b, 50674 Cologne, Germany University of Cologne Cologne Germany

**Keywords:** Chile, endemism, morphology, Patagonia, Subantarctic Forest, taxonomy

## Abstract

*Austrocodrusgladiogeminus***sp. n.** is described from southernmost South America. It is a member of the primitive subfamily Austroserphinae (Hymenoptera, Proctotrupidae), which is distributed in Oceania and South America, and is characterized from other *Austrocodrus* species by its relatively larger body size, the presence of long and sword-shaped ovipositor sheaths, M arising very close and distal to 1cu-a, and Cu and m-cu joining at a distance equivalent to the length of 2cu-a. We consider this species to be a Gondwanan relict. It has southernmost distribution of any proctotrupid.

## Introduction

The parasitic wasp family Proctotrupidae includes about 600 species and 30 genera in two subfamilies: Proctotrupinae and Austroserphinae (= Acanthoserphinae; Townes and Townes 1981). These subfamilies show a strong imbalance in their diversity. The subfamily Austroserphinae, from continental Australia, New Guinea, Tasmania, and southern South America, has three genera with four species, but Proctotrupinae has cosmopolitan distribution and the vast majority part of the family’s diversity (Townes and Townes 1981; Johnson 1992). The first described species of Austroserphinae was *Acanthoserphusalbicoxa* (Dodd 1915), which was described from material collected in Queensland, Australia, and subsequently, Masner in Townes and Townes (1981) described *Acanthoserphusbidens* from Papua New Guinea. Dodd (1933) also described *Austroserphusalbofasciatus*, in a new genus, from material collected in Victoria, Australia. In his revision of Australian proctotrupids, Riek (1955) reported new records for *Austroserphusalbofasciatus* from Tasmania. Ogloblin (1960) described the only South American species known to the date for the subfamily, *Austrocodruspatagonicus*. *Austrocodrus* was originally placed as a subgenus of *Austroserphus* but later raised to the status of genus by Townes and Townes (1981). Ogloblin (1960) described *A.patagonicus* based on specimens collected at Estación Forestal de Pucará, near Lago Lácar, Neuquén, Argentina (40°10'S, 71°27'W). In a revision of material from southernmost Chilean Patagonia, roughly 1700 km south of the type locality of *A.patagonicus*, one specimen belonging to the genus *Austrocodrus* was collected which could not be ascribed to *A.patagonicus*. Based on this material, we describe a second species of *Austrocodrus*, which is the southernmost known proctotrupid.

## Material and methods

The material examined is deposited in the Museo de Zoología de la Universidad de Concepción, Concepción, Chile (MZUCCC 45974). Photographs were made with a Nikon SMZ 745T stereomicroscope and a Nikon D5100 camera using a NII-LED Nikon illuminator. Individual images were stacked with Zerene Stacker Software (2017). The habitus photograph was taken with an AF-S Micro Nikkor 105 mm 1:2.8 G ED lens, and illuminated with Nikon Speedlight SB-800.

The morphological terms and characters follow Townes and Townes (1981), Yoder et al. (2010), and the system of Comstock and Needham (1918) for wing veins and cells and Mason (1986) for vein types. Comparisons between *Austrocodrusgladiogeminus* sp. nov. and *A.patagonicus* were made by reviewing Ogloblin’s (1960) original description, the comments by Townes and Townes (1981), and photographs available at the Proctotrupidae (Hymenoptera) of the World web page (http://proctotrupidae.myspecies.info/taxonomy/term/16), which is managed by Dr Victor Kolyada.

## Results

### Family Proctotrupidae Latreille, 1802

#### Subfamily Austroserphinae Dodd, 1915

##### Genus *Austrocodrus* Ogloblin, 1960

###### Austrocodrus gladiogeminus sp. n.

Taxon classificationAnimaliaHymenopteraProctotrupidae

http://zoobank.org/4BE0E7D9-63B5-4A79-AF29-C90DD8B43287

Figures 1 , 2

####### Type locality.

Chile, Magallanes y de la Antártica Chilena Region, Antártica Chilena Province, Cabo de Hornos County, Puerto Williams City, Parque Etnobotánico Omora (54°56'38"S, 67°39'25"W).

####### Type material.

Holotype: 1 ♀, from the type locality, February 2003. leg. A. Zúñiga. (MZUCCC 45974)

####### Description.

Female. Fore wing length 5.84 mm.

*Head.* Frons with dense pilosity; ocelli globular, translucent. Area between toruli with a marked “Y” shaped carina; the arms of the Y are strongly projected anteriorly. Distance between toruli 0.5 × their diameter (Fig. 2A). Occipital carina absent. Postoccipital carina visible. Maxillary palpus with 4 segments; 2 distalmost segments very elongated. Labial palpus very short, 3-segmented. Mandibles small, not touching each other. Labrum extremely reduced, triangular. Clypeus sharply convex, triangular. Gena in frontal view equal to eye height, strongly excavated to give head a triangular appearance. Scape with conspicuous apical spine, 2× pedicel length. Antenna long, filiform, with 11 antennomeres. The first 2 antennomeres > 10 × as long as wide (Fig. 2C). Eye bare, globular.

**Figure 1.**
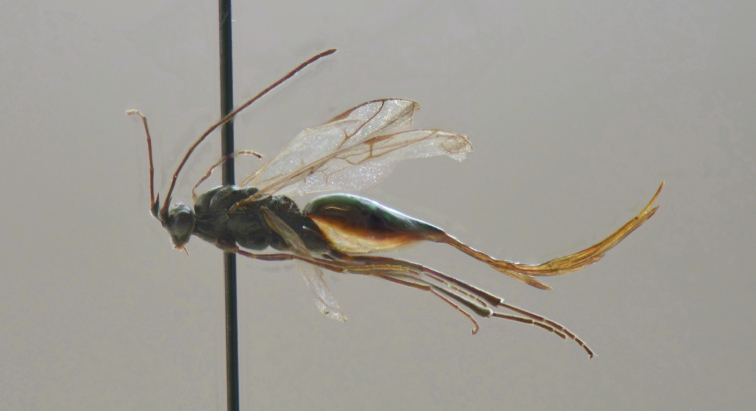
*Austrocodrusgladiogeminus* sp. n. Habitus of female. Holotype MZUCCC 45974.

**Figure 2.**
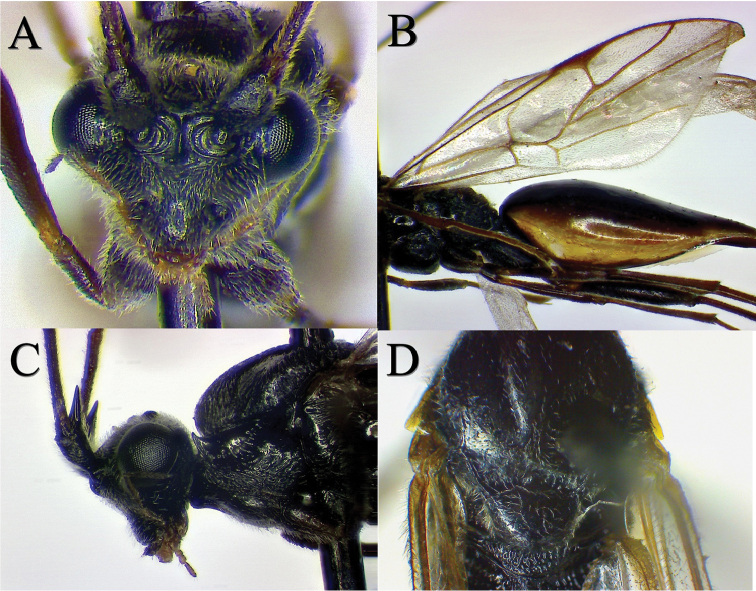
Relevant traits to characterize *Austrocodrusgladiogeminus* sp. n. **A** Head in frontal view showing the “Y” shaped carina between toruli and the triangular outline of the head **B** Fore wing, showing the venation pattern **C** Lateral view showing details from the scape with an apical and conspicuous spine, and the pronotal epomium **D** Dorsal view of mesosoma showing the groove in front of mesoscutellum with 7 foveae.

*Mesosoma.* Pronotum dorsally occluded by mesoscutum and not visible in dorsal view. Epomium strong, which are connected dorsally by carina. First anterodorsal portion of pronotum shiny, smooth, the remainder strongly reticulated, sparsely pilose. Mesoscutum with notauli narrow, deep, originating from the anterior margin not reaching mesoscutellum. Groove in front of mesoscutellum with 7 foveae (Fig. 2D). Scutellum 1.3 × as wide as long, with glabrous center. Axillae deep, sculptured. Metanotum well developed, 1.2 × as long as mesoscutellum, with median section elevated and subtriangular. Axillary trough of metanotum sculptured. Wings hyaline, pilose, with the major part of veins tubular. Anterior portion of M nebulous. Also, M arising close and apical to 1cu-a. Cu and m-cu joining at a distance equal to the length of 2cu-a (Fig. 2B). Propodeum with a short distinct transverse carina on the anterior margin which connects with irregular lateral longitudinal carinae. Petiolar area separated from dorsal propodeum by a strong transverse carina, laterally delimited with low developed apophysis. Propodeum strongly reticulate, pilose and with a raised bare lateral area. Propodeal spiracle elongated, declined 15° from a transverse plane tangent to its ventral end. Propleura flat and strongly pilose. Mesopleuron with prepectus sparsely rugulose and pilose. Prepectal area separated from central mesopleuron by a foveate groove. Central mesopleuron swollen, bare and shiny. Mesopleural suture anteriorly foveate with similar size foveae. Mesepisternum narrow and bare. Mesosternum slightly swollen, strongly pilose. Mesepimeron and mesosternum separated anteriorly by delicate carina anteriorly. Petiole present, with longitudinal wrinkles, 0.3 as long as high.

*Metasoma.* Gaster anteriorly swollen, tapering very strongly to apically. Single synterguite visible with translucent lateral margins. Ovipositor sheaths 2× as long as metasoma and sword-shaped. Ovipositor without apical notch, pointed, only 10% exposed.

**Male.** Unknown.

####### Distribution, habitat and behavior.

The type locality of *A.gladiogeminus* sp. nov. is on the northern coast of Isla Navarino, in the glacially fragmented landscape of southernmost Chile, and lies within the Magellanic Forest biogeographical province in the Subantarctic subregion of the Andean region (sensu Morrone 2015). The new species was collected from a Subantarctic *Nothofagus* forest, dominated by *Nothofaguspumilio* (Poepp. & Endl.) Reiche, and came from a microhabitat of open mixed forest with old and young *N.pumilio* trees. The specimen was collected with a net while flying during an afternoon of the austral summer.

####### Etymology.

The epithet *gladiogeminus* refers to the exceptionally sword-shaped ovipositor sheaths. It is a composition from the Latin noun “gladius”, a sword, the basic weapon of Roman legionnaires after the Punic Wars, and the adjective “geminus”, double or paired.

####### Remarks.

*Austrocodrusgladiogeminus* sp. nov. is the first record of an austroserphine from Chile, and only the second record of the subfamily from South America. Other than the two species of *Austrocodrus*, all other species of the subfamily are distributed in Australia and few southern Pacific islands, which suggests that *Austrocodrus* is a relict of the past connection between these land masses. Additionally, *A.gladiogeminus* sp. nov. has the southernmost distribution of any proctotrupid, reaching almost the 55°S.

The body size of the holotype, with a fore wing length of 5.84 mm, might be the largest among species of the subfamily; the forewing length of *A.patagonicus* is about 3.7 mm, in species of *Acanthoserphus*, between 3.1 and 3.7 mm, and in *Austroserphus* between 5.3 and 5.8 mm. Additionally, we find that the venation pattern of the genus *Austrocodrus* is not so similar to that of *Acanthoserphus* (sensu Townes and Townes 1981). The relative position of M basal to 1cu-a in *Acanthoserphus* and *Austroserphus* allows differentiating these genera from *Austrocodrus*, which has 1cu-a basal to M.

We provide a key to species of the genus *Austrocodrus* (Ogloblin 1960):

**Table:** 

1	M vein tubular, arising apical but not close to 1cu-a. Cu and m-cu joining a short distance in front of 2cu-a. Groove in front of scutellum with 5 foveae. Gaster tapered. Head, mesosoma and metasoma black	***A.patagonicus* Ogloblin**
–	Anterior segment of M vein nebulous. Also, M arising very close and apical to 1cu-a. Cu and m-cu joining at a distance equal to the length of 2cu-a (Figs 1 and 2b). Groove in front of scutellum with 7 foveae (Fig. 2d). Gaster anteriorly swollen tapering very strongly toward posterior apex (Fig. 1). Head and mesosoma black; gaster bicolored, brown at dorsally and creamy white at ventrally (Fig. 1)	***A.gladiogeminus* sp. n. Rodríguez-Serrano and Zúñiga-Reinoso**

## Supplementary Material

XML Treatment for Austrocodrus gladiogeminus
